# The relationship between metabolic syndrome and the incidence of colorectal cancer

**DOI:** 10.1186/s12199-020-00845-w

**Published:** 2020-02-19

**Authors:** JungHyun Lee, Kun Sei Lee, Hyeongsu Kim, Hyoseon Jeong, Min-Jung Choi, Hai-Won Yoo, Tae-Hwa Han, Hyunjung Lee

**Affiliations:** 1 Department of Preventive Medicine, School of Medicine, Konkuk University, Neungdongro 120, Gwangjin-gu, Seoul, 05029 Korea; 2 Health IT Center, College of Medicine, Yonsei University, Seoul, Korea; 3 Department of Nursing, College of Nursing, Konyang University, Daejeon, Korea

**Keywords:** Metabolic syndrome, Colorectal cancer, Relevance, Koreans

## Abstract

**Objectives:**

This study evaluated the incidence of colorectal cancer (CRC) according to the number of metabolic syndrome (MetS) components.

**Methods:**

Using health checkup and insurance claims data of 6,365,409 subjects, the occurrence of CRC according to stage of MetS by sex was determined from the date of the health checkup in 2009 until December 31, 2018.

**Results:**

Cumulative incidence rates (CIR) of CRC in men and women was 3.9 and 2.8 per 1000 (*p* < 0.001), respectively. CIR of CRC for the normal, pre-MetS, and MetS groups in men was 2.6, 3.9, and 5.5 per 1000 (*p* < 0.001) and CIR in women was 2.1, 2.9, and 4.5 per 1000 (*p* < 0.001), respectively. Compared with the normal group, the hazard ratio (HR) of CRC for the pre-MetS group was 1.25 (95% CI 1.17–1.33) in men and 1.09 (95% CI 1.02–1.17) in women, and the HR of CRC for the MetS group was 1.54 (95% CI 1.43–1.65) in men and 1.39 (95% CI 1.26–1.53) in women after adjustment.

**Conclusions:**

We found that MetS is a risk factor for CRC in this study. Therefore, the prevention and active management of MetS would contribute to the prevention of CRC.

## Introduction

Colorectal cancer (CRC) is the third most common cancer worldwide and accounts for 10.2% of all cancers (approximately 1.8 million people a year) [1]. CRC is the second most common cancer after stomach cancer in Korea [1], so identifying and managing risk factors is the first step in preventing CRC. In Korea, the incidence of CRC has increased over the past decade, and the age-adjusted incidence per 100,000 men and women has increased from 26.2, 16.4 in 1999 to 40.4, 22.4 in 2016, respectively [2]. Major risk factors for CRC include genetic predisposition, Western dietary habits, lifestyle (smoking, drinking, physical activity, etc.), and metabolic diseases (obesity, insulin resistance, etc.) [3].

Metabolic syndrome (MetS) is a cluster of metabolic risk factors that includes abdominal obesity, hypertension, hyperglycemia, and dyslipidemia; several definitions have been suggested using different criteria [4, 5]. More than 20% of adults are known to have MetS [6], but its prevalence worldwide varies depending on race, environmental factors, the age and gender composition of the population, genetic differences, physical activity level, eating habits, and differences in measurement standards [7, 8]. The estimated total prevalence of MetS for adults in Korea is 26.9%: 30.0% in males and 24.6% in females [9].

Some studies on the correlation between metabolic syndrome and CRC have been reported recently, but many studies have been conducted in Western countries and races [10–13]. However, studies on Asian races are still limited to East Asian countries such as Taiwan and Japan [14, 15]. In addition, the study population is limited to those who are screened at only university hospital screening centers, so it is not easy to generalize to the entire population [14]. Also there is a limitation that it is difficult to produce a meaningful result because of the short observation period and the lack of cancer cases [15].

In order to complement the limitations of previous research conducted on Asian races, this study evaluated the incidence of CRC according to the number of MetS components using the health checkup data and insurance claims data from the National Health Insurance Service (NHIS) in Korea.

## Methods

### Study design and population

**Fig. 1:**
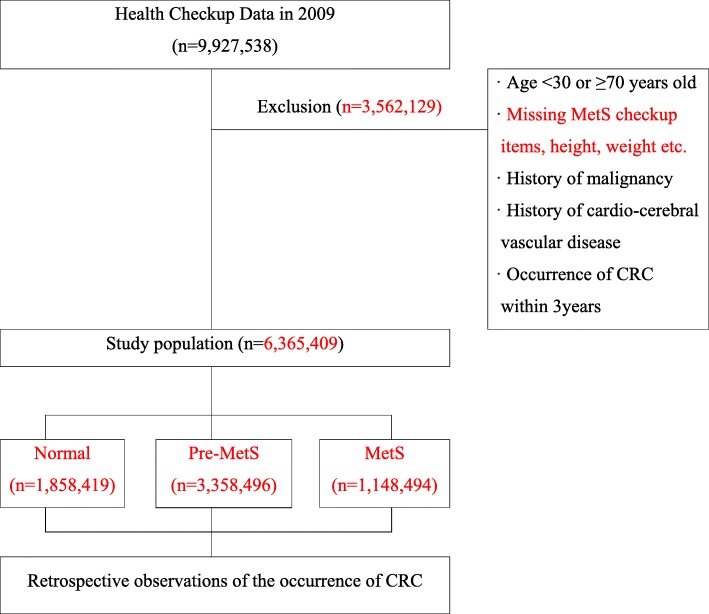
Study design and selection of the study population

This was a retrospective observational study that used health checkup and insurance claims data from the NHIS (Fig. 1). NHIS is constructing and providing health database that includes medical history, treatment, type of disease, and prescription history of all Koreans who use health checkup or medical service under national health insurance. The database has been operating continuously, and about 50 million people’s medical data are monitored from 2002 to 2018. In detail, the National Health Information database is divided into Qualification database, Treatment database, and Health check-up database. The National Health Information database is significant in terms of representation because it monitors medical history of all Koreans, and it is also useful because it can be linked and analyzed with other administrative data through the social security number. The Health check-up database used in this study is very suitable for the purpose of research because it contains the actual measurement value such as blood pressure, blood sugar, triglycerides, HDL, and health habits for those who undergo health checkups every 2 years.

The source population was defined as those who had had health checkups in 2009. While this comprised 15,036,607 people, only 9,927,538 people were actually examined. Of these, 3,562,129 were excluded for the following reasons:

1. Age < 30 years old or ≥ 70 years old.
2. Missing MetS checkup items (fasting blood sugar, systolic and diastolic blood pressure, triglycerides (TG), high-density lipoprotein cholesterol (HDL-C), abdominal obesity); height < 120 or > 200 cm, weight < 20 or > 200 kg.
3. If the subject has a history of cardiovascular disease, cerebrovascular disease, or cancer; cardiovascular diseases (ischemic heart disease I20-I25, cardiac failure I42 or I50), atrial fibrillation (I48), cerebrovascular disease (I60-I69), circulatory system disease (I00-I99), and beneficiaries for cancer (C00-C97).
4. Colorectal cancer found within 3 years after health checkups in 2009.

Consequently, the final study population came to 6,365,409; they were divided into three groups depending on the number of MetS components. There were 1,858,419, 3,358,496, and 1,148,494 in the normal, pre-MetS, and MetS groups, respectively. Using insurance claims data, the occurrence of CRC was checked in the study population from the date of the health checkups in 2009 to December 31, 2018. Mean follow-up period is 9.3 years.

### Measurements

#### Dependent variable

The dependent variable was the occurrence of CRC, as identified operationally from insurance benefit claims data, with codes C18-C20 as a primary diagnosis from the International Classification of Diseases-Tenth revision and V193 as a special code. A special code is a system whereby the economic burden on patients diagnosed with cancer, severe burns, cerebrovascular and heart diseases, or intractable diseases is reduced by decreasing the copayment. If a disease is claimed with this special code, it means that the diagnosis is more accurate than a claim disease without a special code.

#### Independent variable

The independent variable was the stage of MetS, which was classified into three groups according to the criteria of modified National Cholesterol Education Program-Adult Treatment Panel III (NECP-ATP III, which are the most agreed-upon criteria [16]. MetS refers to subjects with at least three of the following five factors: (1) abdominal obesity (waist circumference ≥ 90 cm for men, ≥ 85 cm for women); (2) elevated blood pressure (systolic BP ≥ 130 mmHg or diastolic BP ≥ 85 mmHg or treatment of previously diagnosed hypertension); (3) elevated fasting glucose (≥ 100 mg/dL or treatment of previously diagnosed diabetes mellitus); (4) high TG (≥ 150 mg/dL or drug treatment for high TG); and (5) low HDL-C (< 40 mg/dL for men, < 50 mg/dL for women or drug treatment for low HDL-C). The subjects were placed in the pre-MetS group with one or two MetS factors and the Normal group with no MetS factors.

#### Adjusted variables

The study population was divided into two subsets by gender. Next, age, health behaviors, family history, and laboratory data from the health checkup were used as adjustment variables. Age was divided into 10-year intervals, and health behaviors were selected from smoking, drinking, and physical activities in the questionnaire. Smoking was categorized into three groups: never smoked, smoked in the past but no longer, and currently smoking. Alcohol consumption was categorized into four groups based on the drinking frequency: non-drinker, 2–3 times per month, 1–4 times per week, and almost every day. Physical activity group was categorized into three groups based on frequency: never, 1–4 times per week, and almost every day. Family history of cancer was divided into two groups of yes or no. Height, weight, body mass index (BMI), hemoglobin, serum creatinine, total cholesterol, and alanine aminotransferase (ALT), which were health checkup items, were used as laboratory findings, and as continuous variables. BMI (kg/m^2^), hemoglobin (g/dL), and serum creatinine (mg/dL) are categorized according to the criteria of Korean health screening [17]. Total cholesterol (mg/dL) and ALT (IU/L) were used in the model as continuous variables, and the original data values were increased by ten times to improve the readability of the hazard ratio.

Height (cm) was divided into four groups according to the quartiles of height distribution for each sex (< 167, 167–171, 172–174, ≥ 175 cm for men; < 154, 154–156, 157–160, ≥ 161 cm for women). Weight (kg) also was divided into four groups according to the quartiles of weight distribution for each sex (< 64, 64–69, 70–76, ≥ 77 cm for men; < 52, 52–55, 56–61, ≥ 62 kg for women). BMI was categorized into five groups: underweight (< 18.5), normal (18.5–22.9), overweight (23.0–24.9), obesity (25–29.9), and altitude obesity (≥ 30). Hemoglobin was categorized into three groups and differed by sex: normal (men: > 12.0, women: > 10.0), mild or moderate anemia (men: 10.0–12.0, women: 8.0–10.0), severe anemia (men: < 10.0, women: < 8); serum creatinine was categorized into two groups: normal (<1.5), abnormal (≥ 1.5). Total cholesterol and ALT were also divided into four groups for according to the quartiles of their distribution for each sex (total cholesterol < 173, 173–194, 195–218, ≥ 219, ALT < 18, 18–24, 25–35, ≥ 36 for men; total cholesterol < 171, 171–192, 193–217, ≥ 218, ALT < 13, 13–15, 16–21, ≥ 22 for women) for descriptive analysis.

### Statistical analysis

We summarized the frequency of study population for related variables according to the number of MetS components, and calculated the cumulative incidence rate (CIR) and incidence density (ID) of CRC. CIR is the incidence per 1000 person and ID is the incidence per 10,000 person-years (PY). PY was calculated as the time from the baseline examination to the date of CRC diagnosis, the date of death, or December 31, 2018 when who survives without diagnosis of CRC.

Next, we compared the frequency between variables using the chi-square test. To analyze the risk of developing CRC due to MetS, Cox proportional hazard regression was applied. All variables affecting the incidence of CRC were considered stepwise and five Cox proportional hazard models were fitted, including one unadjusted model for comparison.

Model 1 was adjusted for age. Model 2 was adjusted for health behavior (smoking, exercise). Model 3 was adjusted for family history cancer. Model 4 was adjusted for the laboratory findings (height, weight, hemoglobin, and other relevant values). Model 5 was adjusted for the laboratory findings (body mass index, creatinine, hemoglobin, and other relevant values). The results are summarized as the hazard ratio (HR) and 95% confidence interval (95% CI).

A level of α = 0.05 was used to determine the significance of the models and variables. All statistical analyses were performed using SAS ver. 9.1 (SAS Institute, Cary, NC, USA).

### Ethics considerations

The study was approved by the institutional review board of Konkuk University (7001355-201909-E-100).

## Results

### CIR and ID of CRC according to the number of Mets components in men

The number of study population and the CIR of CRC in men were 3,695,923 and 3.9 (Table 1). The CIR of CRC by MetS stage was 2.6, 3.9, and 5.5 for the normal, pre-MetS, and MetS groups, respectively (*p* < 0.001).

**Table 1:** Cumulative incidence rate and incidence density of colorectal cancer according to the progression of metabolic syndrome in men

Characteristics	Category	No. of study population				No. of colorectal cancer				Cumulative incidence rate (per 1000)						Incidence density (per 10,000 person-years)					
		Normal	Pre-MetS	MetS	Total	Normal	Pre-MetS	MetS	Total	Normal	Pre-MetS	MetS	*p* value	Total	*p* value	Normal	Pre-MetS	MetS	*p* value	Total	*p* value
Total		841,887	2,037,811	816,225	3,695,923	2174	7858	4517	14,549	2.6	3.9	5.5		3.9		2.8	4.2	6.0		4.2	
Age	30–39	52,090	78,689	19,451	150,230	31	62	11	104	0.6	0.8	0.6	< 0.001	0.7	< 0.001	0.6	0.8	0.6	< 0.001	0.7	< 0.001
	40–49	403,228	802,049	267,504	1,472,781	457	1066	422	1945	1.1	1.3	1.6	< 0.001	1.3		1.2	1.4	1.7	< 0.001	1.4	
	50–59	259,358	695,206	294,199	1,248,763	771	2722	1387	4880	3.0	3.9	4.7	< 0.001	3.9		3.2	4.2	5.1	< 0.001	4.2	
	60–69	127,211	461,867	235,071	824,149	915	4008	2697	7620	7.2	8.7	11.5	< 0.001	9.2		7.8	9.4	12.5	< 0.001	10.0	
Smoking	Non-smoker	254,976	551,062	198,274	1,004,312	599	1998	1098	3695	2.3	3.6	5.5	< 0.001	3.7	<0.001	2.5	3.9	6.0	< 0.001	4.0	< 0.001
	Ex-smoker	191,807	502,695	209,573	904,075	506	2112	1330	3948	2.6	4.2	6.3	< 0.001	4.4		2.8	4.5	6.8	< 0.001	4.7	
	Smoker	390,833	975,701	405,252	1,771,786	1061	3718	2076	6855	2.7	3.8	5.1	< 0.001	3.9		2.9	4.1	5.6	< 0.001	4.2	
Alcohol consumption	No drink	266,110	561,954	205,282	1,033,346	722	2099	1121	3942	2.7	3.7	5.5	< 0.001	3.8	< 0.001	2.9	4.0	5.9	< 0.001	4.1	< 0.001
	2–3/per month	435,666	1,015,825	391,247	1,842,738	965	3469	1882	6316	2.2	3.4	4.8	< 0.001	3.4		2.4	3.7	5.2	< 0.001	3.7	
	1–4/per week	100,885	330,563	157,013	588,461	309	1479	984	2772	3.1	4.5	6.3	< 0.001	4.7		3.3	4.8	6.8	< 0.001	5.1	
	5/per week	29,429	110,628	56,183	196,240	149	745	503	1397	5.1	6.7	9.0	< 0.001	7.1		5.5	7.3	9.8	< 0.001	7.7	
Physical exercise, per week	No exercise	343,189	841,263	346,095	1,530,547	897	3244	1948	6089	2.6	3.9	5.6	< 0.001	4.0	< 0.001	2.8	4.2	6.1	< 0.001	4.3	< 0.001
	1–4/per week	177,100	419,415	167,585	764,100	452	1489	854	2795	2.6	3.6	5.1	< 0.001	3.7		2.7	3.8	5.5	< 0.001	3.9	
	5/per week	314,767	762,500	297,226	1,374,493	807	3072	1694	5573	2.6	4.0	5.7	< 0.001	4.1		2.7	4.3	6.2	< 0.001	4.4	
FHx of cancer	No	494,125	1,199,780	486,757	2,180,662	1,176	4376	2520	8072	2.4	3.6	5.2	< 0.001	3.7	< 0.001	2.6	3.9	5.6	< 0.001	4.0	< 0.001
	Yes	95,222	217,852	83,206	396,280	234	816	467	1517	2.5	3.7	5.6	< 0.001	3.8		2.6	4.0	6.1	< 0.001	4.1	
Height (cm)	< 167	239,064	636,453	236,248	1,111,765	822	3205	1723	5750	3.4	5.0	7.3	< 0.001	5.2	< 0.001	3.7	5.4	7.2	< 0.001	5.6	< 0.001
	167–171	220,547	534,838	209,648	965,033	588	2131	1193	3912	2.7	4.0	5.7	< 0.001	4.1		2.9	4.3	5.6	< 0.001	4.4	
	172-174	204,006	469,535	192,522	866,063	435	1485	950	2870	2.1	3.2	4.9	< 0.001	3.3		2.3	3.4	4.8	< 0.001	3.6	
	≥ 175	178,270	396,985	177,807	753,062	329	1037	651	2017	1.8	2.6	3.7	< 0.001	2.7		2.0	2.8	3.6	< 0.001	2.9	
Weight (kg)	< 64	372,420	572,899	83,536	1,028,855	1,106	2,672	646	4,424	3.0	4.7	7.7	< 0.001	4.3	< 0.001	3.2	5.0	8.5	< 0.001	4.6	< 0.001
	64-69	235,461	530,335	128,262	894,058	555	2157	871	3583	2.4	4.1	6.8	< 0.001	4.0		2.5	4.4	7.4	< 0.001	4.3	
	70–76	171,877	534,264	213,956	920,097	397	1852	1277	3526	2.3	3.5	6.0	< 0.001	3.8		2.5	3.7	6.4	< 0.001	4.1	
	≥ 77	62,129	400,313	390,471	852,913	116	1177	1723	3016	1.9	2.9	4.4	< 0.001	3.5		2.0	3.2	4.8	< 0.001	3.8	
BMI (kg/m^2^)	< 18.5	33,171	29,275	1,844	64,290	76	144	21	241	2.3	4.9	11.4	< 0.001	3.7	< 0.001	2.5	5.4	13.1	< 0.001	4.1	< 0.001
	18.5–22.9	445,140	656,729	83,735	1,185,604	1,138	2617	572	4327	2.6	4.0	6.8	< 0.001	3.6		2.7	4.3	7.4	< 0.001	3.9	
	23.0–24.9	234,677	621,458	159,332	1,015,467	631	2384	977	3992	2.7	3.8	6.1	< 0.001	3.9		2.9	4.1	6.6	< 0.001	4.2	
	25.0–29.9	128,038	690,021	477,677	1,295,736	327	2580	2537	5444	2.6	3.7	5.3	< 0.001	4.2		2.7	4.0	5.7	< 0.001	4.5	
	≥ 30.0	861	40,328	93,637	134,826	2	133	410	545	2.3	3.3	4.4	< 0.001	4.0		2.5	3.6	4.7	< 0.001	4.4	
Total cholesterol (mg/dL)	< 173	287,648	499,111	154,244	941,003	650	1819	886	3355	2.3	3.6	5.7	< 0.001	3.6	< 0.001	2.4	3.9	6.2	< 0.001	3.8	< 0.001
	173–194	239,234	504,393	176,051	919,678	615	1886	961	3462	2.6	3.7	5.5	< 0.001	3.8		2.8	4.0	5.9	< 0.001	4.1	
	195–218	189,771	516,797	210,313	916,881	530	2,044	1,159	3,733	2.8	4.0	5.5	< 0.001	4.1		3.0	4.3	6.0	< 0.001	4.4	
	≥ 219	125,234	517,510	275,617	918,361	379	2,109	1,511	3,999	3.0	4.1	5.5	< 0.001	4.4		3.3	4.4	5.9	< 0.001	4.7	
ALT (IU/L)	< 18	354,952	530,427	94,775	980,154	880	2,154	628	3,662	2.5	4.1	6.6	< 0.001	3.7	< 0.001	2.7	4.4	7.2	< 0.001	4.0	< 0.001
	18–24	241,797	549,667	159,960	951,424	648	2,144	948	3,740	2.7	3.9	5.9	< 0.001	3.9		2.9	4.2	6.4	< 0.001	4.2	
	25–35	154,775	500,574	221,678	877,027	417	1967	1277	3661	2.7	3.9	5.8	< 0.001	4.2		2.9	4.2	6.2	< 0.001	4.5	
	≥ 36	90,363	457,143	339,812	887,318	229	1593	1664	3486	2.5	3.5	4.9	< 0.001	3.9		2.7	3.8	5.3	< 0.001	4.2	
Hemoglobin (g/dL)	> 12	836,812	2,025,415	811,497	3,673,724	2,153	7,772	4,476	14,401	2.6	3.8	5.5	< 0.001	3.9	< 0.001	2.8	4.1	6.0	< 0.001	4.2	< 0.001
	10–12	4,173	9,967	3,769	17,909	15	64	31	110	3.6	6.4	8.2	< 0.001	6.1		3.9	7.2	9.4	< 0.001	6.9	
	< 10	624	1,624	623	2,871	5	17	6	28	8.0	10.5	9.6	< 0.001	9.8		8.8	11.9	11.2	< 0.001	11.1	
Serum creatinine (mg/dL)	≤ 1.5	815,023	1,962,815	783,300	3,561,138	2,112	7599	4339	14,050	2.6	3.9	5.5	< 0.001	3.9	< 0.001	2.8	4.2	6.0	< 0.001	4.3	< 0.001
	> 1.5	26,813	74,886	32,883	134,582	61	259	178	498	2.3	3.5	5.4	< 0.001	3.7		2.4	3.7	5.8	< 0.001	4.0	

*MetS* metabolic syndrome, *FHx* family history, *BMI* body mass index, *ALT* alanine aminotransferase

The ID of CRC in men was 4.2 (Table 1). The ID of CRC by MetS stage was 2.8, 4.2, and 6.0 for the normal, pre-MetS, and MetS groups, respectively (*p* < 0.001).

The number of study population and the CIR and the ID of CRC for the other variables in men were summarized in Table 1.

### CIR and ID of CRC according to the number of Mets components in women

The number of study population and the CIR of CRC in women were 2,669,486 and 2.8 (Table 2). The CIR of CRC by MetS stage was 2.1, 2.9, and 4.5 for the normal, pre-MetS, and MetS groups, respectively (*p* < 0.001).

**Table 2:** Cumulative incidence rate and incidence density of colorectal cancer according to the progression of metabolic syndrome in women

Characteristics	Category	No. of study population				No. of colorectal cancer				Cumulative incidence rate (per 1000)						Incidence density (per 10,000 person-years)					
		Normal	Pre-MetS	MetS	Total	Normal	Pre-MetS	MetS	Total	Normal	Pre-MetS	MetS	*p* value	Total	*p* value	Normal	Pre-MetS	MetS	*p* value	Total	*p* value
Total		1,016,532	1,320,685	332,269	2,669,486	2119	3827	1493	7439	2.1	2.9	4.5		2.8		2.2	3.1	4.8		3.0	
Age	30–39	44,462	24,310	1909	70,681	29	14	1	44	0.7	0.6	0.5	< 0.001	0.6	< 0.001	0.7	0.6	0.6	< 0.001	0.7	< 0.001
	40–49	355,151	277,454	33,864	666,469	363	323	46	732	1.0	1.2	1.4	< 0.001	1.1		1.1	1.3	1.5	< 0.001	1.2	
	50–59	424,813	555,494	114,030	1,094,337	1030	1464	416	2910	2.4	2.6	3.6	< 0.001	2.7		2.6	2.8	3.9	< 0.001	2.9	
	60–69	192,106	463,427	182,466	837,999	697	2026	1030	3753	3.6	4.4	5.6	< 0.001	4.5		3.9	4.7	6.0	< 0.001	4.8	
Smoking	Non-smoker	958,586	1,249,613	312,149	2,520,348	1,997	3581	1393	6971	2.1	2.9	4.5	< 0.001	2.8	< 0.001	2.2	3.1	4.8	< 0.001	3.0	< 0.001
	Ex-smoker	20,223	21,067	5,102	46,392	45	69	23	137	2.2	3.3	4.5	< 0.001	3.0		2.4	3.5	4.8	< 0.001	3.2	
	Smoker	31,594	42,661	13,015	87,270	71	155	68	294	2.2	3.6	5.2	< 0.001	3.4		2.4	3.9	5.6	< 0.001	3.6	
Alcohol consumption	No drink	695,610	956,081	255,189	1,906,880	1,460	2792	1167	5419	2.1	2.9	4.6	< 0.001	2.8	< 0.001	2.3	3.1	4.9	< 0.001	3.0	< 0.001
	2–3/per month	267,878	291,847	58,767	618,492	549	816	245	1610	2.0	2.8	4.2	< 0.001	2.6		2.2	3.0	4.5	< 0.001	2.8	
	1–4/per week	30,509	41,508	9,949	81,966	76	132	42	250	2.5	3.2	4.2	< 0.001	3.1		2.7	3.4	4.5	< 0.001	3.3	
	5/per week	8,547	13,865	3,936	26,348	18	42	17	77	2.1	3.0	4.3	< 0.001	2.9		2.3	3.3	4.6	< 0.001	3.1	
Physical exercise, per week	No exercise	529,972	692,901	179,742	1,402,615	1092	1998	781	3871	2.1	2.9	4.3	< 0.001	2.8	< 0.001	2.2	3.1	4.7	< 0.001	3.0	< 0.001
	1–4/per week	191,856	241,698	59,221	492,775	412	721	270	1403	2.1	3.0	4.6	< 0.001	2.8		2.3	3.2	4.9	< 0.001	3.1	
	5/per week	288,778	379,098	91,511	759,387	611	1092	432	2135	2.1	2.9	4.7	< 0.001	2.8		2.3	3.1	5.1	< 0.001	3.0	
FHx of cancer	No	554,083	725,060	189,415	1,468,558	1,103	2069	854	4026	2.0	2.9	4.5	< 0.001	2.7	< 0.001	2.1	3.1	4.8	< 0.001	2.9	< 0.001
	Yes	121,122	147,564	34,493	303,179	297	441	174	912	2.5	3.0	5.0	< 0.001	3.0		2.6	3.2	5.4	< 0.001	3.2	
Height (cm)	<154	264,445	431,540	124,997	820,982	628	1419	613	2657	2.4	1.5	4.9	< 0.001	3.2	< 0.001	2.5	3.5	5.2	< 0.001	3.5	< 0.001
	154–156	210,014	285,243	73,169	568,426	458	873	360	1691	2.7	4.0	5.7	< 0.001	4.1		2.3	3.3	5.3	< 0.001	3.2	
	157–160	285,846	344,154	80,677	710,677	606	946	302	1854	2.1	3.2	4.9	< 0.001	3.3		2.3	2.9	4.0	< 0.001	2.8	
	≥ 161	256,227	259,748	53,426	569,401	427	589	218	1234	1.8	2.6	3.7	< 0.001	2.7		1.8	2.4	4.4	< 0.001	2.3	
Weight (kg)	< 52	413,406	340,565	29,049	783,020	770	943	121	1834	1.9	2.3	4.2	< 0.001	2.3	< 0.001	2.0	3.0	4.5	< 0.001	2.5	<0.001
	52–55	255,743	285,512	38,948	580,203	542	806	174	1522	2.1	1.9	4.5	< 0.001	2.6		2.3	3.0	4.8	< 0.001	2.8	
	56–61	245,247	375,870	83,951	705,068	578	1127	377	2082	2.4	1.5	4.5	< 0.001	3.0		2.5	3.2	4.8	< 0.001	3.2	
	≥ 62	102,136	318,738	180,321	601,195	229	951	821	2001	2.2	0.7	4.6	< 0.001	3.3		2.4	3.2	4.9	< 0.001	3.6	
BMI (kg/m^2^)	< 18.5	69,872	34,585	1,015	105,472	113	69	4	186	1.6	3.3	3.9	< 0.001	1.8	< 0.001	1.7	2.1	4.3	< 0.001	1.9	< 0.001
	18.5–22.9	650,264	583,930	51,625	1,285,819	1,270	1518	203	2991	2.0	2.2	3.9	< 0.001	2.3		2.1	2.8	4.2	< 0.001	2.5	
	23.0-24.9	199,271	340,173	69,405	608,849	488	1055	309	1852	2.4	1.4	4.5	< 0.001	3.0		2.6	3.3	4.8	< 0.001	3.3	
	25.0–29.9	95,519	328,761	164,397	588,677	242	1083	770	2095	2.5	0.7	4.7	< 0.001	3.6		2.7	3.5	5.0	< 0.001	3.8	
	≥ 30.0	1,606	33,236	45,827	80,669	6	102	207	315	3.7	0.2	4.5	< 0.001	3.9		4.0	3.3	4.8	< 0.001	4.2	
Total cholesterol (mg/dL)	<171	297,122	339,711	56,297	693,130	506	699	218	1423	1.7	1.5	3.9	< 0.001	2.1	< 0.001	1.8	2.2	4.2	< 0.001	2.2	< 0.001
	171-192	285,233	308,510	65,401	659,144	528	852	289	1669	1.9	1.7	4.4	< 0.001	2.5		2.0	3.0	4.7	< 0.001	2.7	
	193–217	250,903	323,283	84,889	659,075	598	1033	382	2013	2.4	1.8	4.5	< 0.001	3.1		2.6	3.4	4.8	< 0.001	3.3	
	≥ 218	183,274	349,181	125,682	658,137	487	1243	604	2334	2.7	1.4	4.8	< 0.001	3.5		2.8	3.8	5.2	< 0.001	3.8	
ALT (IU/L)	<13	414,706	370,487	39,262	824,455	752	884	158	1794	1.8	2.0	4.0	< 0.001	2.2	< 0.001	1.9	2.6	4.3	< 0.001	2.3	< 0.001
	13–15	219,168	259,979	43,677	522,824	453	754	183	1390	2.1	1.7	4.2	< 0.001	2.7		2.2	3.1	4.5	< 0.001	2.8	
	16-21	230,941	354,345	88,877	674,163	554	1094	407	2055	2.4	1.6	4.6	< 0.001	3.0		2.6	3.3	4.9	< 0.001	3.3	
	≥ 22	151,717	335,874	160,453	648,044	360	1095	745	2200	2.4	1.1	4.6	< 0.001	3.4		2.5	3.5	5.0	< 0.001	3.6	
Hemoglobin (g/dL)	> 10	986,791	1,281,381	325,615	2,593,787	2,060	3742	1464	7266	2.1	1.6	4.5	< 0.001	2.8	< 0.001	2.2	3.1	4.8	< 0.001	3.0	< 0.001
	8–10	25,669	32,557	5,620	63,846	51	74	25	150	2.0	1.6	4.4	< 0.001	2.3		2.1	2.4	4.8	< 0.001	2.5	
	< 8	4,005	6,650	1,005	11,660	8	11	4	23	2.0	1.2	4.0	< 0.001	2.0		2.1	1.8	4.3	< 0.001	2.1	
Serum creatinine (mg/dL)	≤ 1.5	999,732	1,298,976	326,608	2,625,316	2,090	3774	1466	7330	2.1	1.6	4.5	< 0.001	2.8	< 0.001	2.2	3.1	4.8	< 0.001	3.0	< 0.001
	> 1.5	16,732	21,632	5,649	44,013	29	53	27	109	1.7	1.3	4.8	< 0.001	2.5		1.9	2.6	5.1	< 0.001	2.7	

*MetS* metabolic syndrome, *FHx* family history, *BMI* body mass index, *ALT* alanine aminotransferase

The ID of CRC in women was 3.0 (Table 2). The ID of CRC by MetS stage was 2.2, 3.1, and 4.8 for the normal, pre-MetS, and MetS groups, respectively (*p* < 0.001).

The number of study population and the CIR and the ID of CRC for the other variables in women were summarized in Table 2.

### Risk of the number of Mets components on CRC in men and women

In men, the HR of CRC for the pre-MetS group compared with the normal group before adjustment (unadjusted model) was 1.50 (95% CI 1.43–1.57), and the HR for the MetS group was 2.16 (95% CI 2.06–2.28) (Table 3). After full adjustment (model 5), the HR for the pre-MetS group and MetS group was 1.25 (95% CI 1.17–1.33) and 1.54 (95% CI 1.43–1.65).

**Table 3:** Hazard ratios of the cumulative incidence rate of colorectal cancer according to the progression of metabolic syndrome in men

Characteristics	Category	HR (95% CI)					
		Non–Adjusted	^a^Model 1	^b^Model 2	^c^Model 3	^d^Model 4	^e^Model 5
Metabolic syndrome (MetS) stage	Normal	Ref.	Ref.	Ref.	Ref.	Ref.	Ref
	Pre-MetS	1.50 (1.43 1.57)	1.24 (1.19 1.30)	1.22 (1.17 1.28)	1.26 (1.19 1.34)	1.24 (1.17 1.32)	1.25 (1.17 1.33)
	MetS	2.16 (2.06 2.28)	1.58 (1.50 1.66)	1.54 (1.46 1.62)	1.59 (1.49 1.70)	1.54 (1.43 1.65)	1.54 (1.43 1.65)
Age	30–39		Ref.	Ref.	Ref.	Ref.	Ref.
	40–49		1.86 (1.53 2.26)	1.85 (1.52 2.26)	1.88 (1.48 2.39)	1.88 (1.48 2.40)	1.88 (1.48 2.40)
	50–59		5.36 (4.42 6.52)	5.35 (4.39 6.51)	5.31 (4.19 6.73)	5.31 (4.20 6.74)	5.35 (4.22 6.79)
	60		12.49 (10.29 15.16)	12.48 (10.26 15.18)	12.48 (9.85 15.80)	12.52 (9.87 15.89)	12.67 (10.00 16.06)
Smoking	Non-smoker			Ref.	Ref.	Ref.	Ref.
	Ex-smoker			1.05 (1.00 1.10)	1.07 (1.01 1.13)	1.07 (1.01 1.13)	1.07 (1.01 1.13)
	Smoker			1.17 (1.12 1.22)	1.19 (1.13 1.26)	1.19 (1.13 1.26)	1.19 (1.13 1.25)
Alcohol consumption	No drink			Ref.	Ref.	Ref.	Ref.
	2–3/per month			1.15 (1.01 1.10)	1.05 (1.00 1.10)	1.05 (1.00 1.11)	1.05 (1.00 1.11)
	1–4/per week			1.19 (1.13 1.25)	1.16 (1.09 1.24)	1.16 (1.10 1.24)	1.16 (1.10 1.24)
	5/per week			1.38 (1.29 1.47)	1.40 (1.29 1.51)	1.39 (1.29 1.51)	1.39 (1.29 1.51)
Physical exercise, per week	No exercise			Ref.	Ref.	Ref.	Ref.
	1–4/per week			0.97 (0.93 1.02)	0.97 (0.97 1.02)	0.97 (0.92 1.03)	0.97 (0.92 1.03)
	5/per week			0.97 (0.94 1.01)	0.98 (0.94 1.03)	0.99 (0.94 1.04)	0.99 (0.94 1.04)
FHx of cancer	No				Ref.	Ref.	Ref.
	Yes				0.99 (0.93 1.05)	0.99 (0.93 1.05)	0.99 (0.93 1.04)
Hemoglobin (g/dL)	> 12					Ref	Ref
	10–12					1.24 (0.97 1.60)	1.24 (0.97 1.59)
	< 10					2.03 (1.24 3.30)	2.02 (1.24 3.30)
Serum creatinine (mg/dL)	≤ 1.5					Ref	Ref
	> 1.5					0.92 (0.84 1.02)	0.92 (0.84 1.02)
^f^Total cholesterol (mg/dL)						1.02 (1.01 1.03)	1.02 (1.01 1.03)
^f^ALT (IU/L)						1.01 (1.00 1.02)	1.01 (1.00 1.02)
Height (cm)	< 167					Ref	
	167–171					0.98 (0.93 1.04)	
	172-174					0.95 (0.89 1.01)	
	≥ 175					0.97 (0.90 1.05)	
Weight (kg)	< 64					Ref	
	64–69					1.00 (0.94 1.05)	
	70–76					0.97 (0.91 1.03)	
	≥77					1.05 (0.98 1.13)	
BMI (kg/m^2^)	< 18.5						1.11 (0.94 1.32)
	18.5–22.9						Ref.
	23.0–24.9						0.98 (0.93 1.03)
	25.0–29.9						1.00 (0.95 1.06)
	≥ 30.0						1.16 (1.04 1.30)

Values are presented as β (95% confidence interval)

*MetS* metabolic syndrome, *Ref* reference, *FHx* family history, *ALT* alanine aminotransferase, *BMI* body mass index

^a^Model 1: adjusted for age

^b^Model 2: adjusted for age, smoking, alcohol consumption, and exercise

^c^Model 3: adjusted for age, smoking, alcohol consumption, exercise, FHx of cancer

^d^Model 4: adjusted for age, smoking, alcohol consumption, exercise, FHx of cancer, hemoglobin, serum creatinine, total cholesterol and ALT, hight, weight

^e^Model 5: adjusted for age, smoking, alcohol consumption, exercise, FHx of cancer, hemoglobin, serum creatinine, total cholesterol and ALT, BMI

^f^Total cholesterol, ^f^ALT: These continuous variables were analyzed by increasing 10 units in the original data to improve the readability of the hazard ratio analysis

In women, the HR of CRC for the pre-MetS group compared with the normal group before adjustment (unadjusted model) was 1.39 (95% CI 1.32–1.46), and the HR for the MetS group was 2.15 (95% CI 2.01–2.30) (Table 4). After full adjustment (model 5), the HR for the pre-MetS group and MetS group was 1.09 (95% CI 1.02–1.17) and 1.39 (95% CI 1.26–1.53).

**Table 4:** Hazard ratios of the cumulative incidence rate of colorectal cancer according to the progression of metabolic syndrome in women

Characteristics	Category	HR (95% CI)					
		Non–Adjusted	^a^Model 1	^b^Model 2	^c^Model 3	^d^Model 4	^e^Model 5
Metabolic syndrome (MetS) stage	Normal	Ref.	Ref.	Ref.	Ref.	Ref	Ref.
	Pre-MetS	1.39 (1.32 1.46)	1.14 (1.08 1.20)	1.13 (1.07 1.19)	1.12 (1.05 1.20)	1.09 (1.01 1.16)	1.09 (1.02 1.17)
	MetS	2.15 (2.01 2.30)	1.50 (1.40 1.61)	1.49 (1.39 1.59)	1.52 (1.40 1.66)	1.39 (1.26 1.52)	1.39 (1.26 1.53)
Age	30-39		Ref.	Ref.	Ref.	Ref.	Ref.
	40-49		1.74 (1.28 2.36)	1.77 (1.30 2.41)	1.66 (1.15 2.40)	1.62 (1.12 2.34)	1.64 (1.13 2.37)
	50-59		4.03 (2.99 5.43)	4.19 (3.10 5.66)	3.89 (2.71 5.59)	3.71 (2.58 5.33)	3.76 (2.62 5.40)
	60		6.38 (4.74 8.60)	6.74 (4.99 9.12)	6.37 (4.44 9.15)	5.97 (4.15 8.60)	6.04 (4.20 8.68)
Smoking	Non-smoker			Ref.	Ref.	Ref.	Ref.
	Ex-smoker			1.25 (1.06 1.49)	1.32 (1.08 1.60)	1.31 (1.07 1.59)	1.32 (1.08 1.60)
	Smoker			1.25 (1.10 1.41)	1.23 (1.06 1.43)	1.23 (1.06 1.43)	1.23 (1.06 1.43)
Alcohol consumption	No drink			Ref.	Ref.	Ref.	Ref.
	2–3/per month			1.11 (1.05 1.18)	1.12 (1.05 1.21)	1.12 (1.05 1.21)	1.12 (1.05 1.20)
	1–4/per week			1.20 (1.05 1.36)	1.13 (0.96 1.33)	1.13 (0.96 1.33)	1.13 (0.96 1.33)
	5/per week			0.99 (0.79 1.24)	1.07 (0.82 1.40)	1.07 (0.82 1.40)	1.07 (0.82 1.40)
Physical exercise, per week	No exercise			Ref.	Ref.	Ref.	Ref.
	1–4/per week			1.04 (0.98 1.10)	1.03 (0.96 1.11)	1.03 (0.96 1.11)	1.03 (0.96 1.12)
	5/per week			0.96 (0.91 1.01)	0.96 (0.89 1.02)	0.95 (0.89 1.02)	0.96 (0.90 1.02)
FHx of cancer	No				Ref.	Ref.	Ref.
	Yes				1.09 (1.01 1.17)	1.09 (1.01 1.17)	1.09 (1.01 1.17)
Hemoglobin (g/dL)	> 10					Ref	Ref
	8–10					1.15 (0.94 1.40)	1.15 (0.94 1.40)
	< 8					0.91 (0.55 1.51)	0.90 (0.54 1.50)
Serum creatinine (mg/dL)	≤ 1.5					Ref	Ref
	> 1.5					0.92 (0.75 1.13)	0.92 (0.75 1.13)
^e^Total cholesterol(mg/dL)						1.03 (1.01 1.04)	1.03 (1.01 1.04)
^f^ALT (IU/L)						1.00 (0.99 1.02)	1.00 (0.99 1.02)
Height (cm)	< 154					Ref	
	154–156					1.05 (0.97 1.13)	
	157–160					0.91 (0.88 1.03)	
	≥161					0.94 (0.85 1.03)	
Weight (kg)	< 52					Ref	
	52–55					1.03 (0.94 1.12)	
	56–61					1.09 (1.01 1.19)	
	≥ 62					1.18 (1.08 1.29)	
^e^BMI (kg/m^2^)	< 18.5						1.01 (0.84 1.22)
	18.5–22.9						Ref
	23.0–24.9						1.09 (1.01 1.17)
	25.0–29.9						1.13 (1.05 1.22)
	≥ 30.0						1.20 (1.03 1.39)

Values are presented as β (95% confidence interval)

*MetS* metabolic syndrome, *Ref* reference, *FHx* family history, *ALT* alanine aminotransferase, *BMI* body mass index

^a^Model 1: adjusted for age

^b^Model 2: adjusted for age, smoking, alcohol consumption, and exercise

^c^Model 3: adjusted for age, smoking, alcohol consumption, exercise, FHx of cancer

^d^Model 4: adjusted for age, smoking, alcohol consumption, exercise, FHx of cancer, hemoglobin, serum creatinine, total cholesterol and ALT, hight, weight

^e^Model 5: adjusted for age, smoking, alcohol consumption, exercise, FHx of cancer, hemoglobin, serum creatinine, total cholesterol and ALT, BMI

^f^Total cholesterol, ^f^ALT: These continuous variables were analyzed by increasing 10 units in the original data to improve the readability of the hazard ratio analysis

## Discussion

To investigate the effect of MetS on the incidence of CRC, we analyzed about 6 million medical claim and check-up data that had the high external validity with large sample size and average follow-up period of 9.3 years per person from NHIS in Korea.

The results of the study revealed there is a positive association between MetS and the incidence of CRC. This supports previous findings that MetS is the main factor expediting tumor growth [18–20].

After analyzing 18 studies (687,413 people) of MetS and CRC, Jinjuvadia et al. [18] reported that MetS increases the occurrence of CRC [relative rate (RR), 1.30; 95% CI 1.18–1.43] and colorectal adenoma (RR, 1.37; 95% CI 1.26–1.494). The Atherosclerosis Risk in Communities (ARIC) follow-up observational study of 14,000 Americans identified MetS as the main factor (RR, 1.49; 95% CI 1.0–2.4) responsible for the occurrence of CRC [19]. With 12 years of follow-up, the Metabolic Syndrome and Cancer Project determined that the risk of CRC in men and women increased by 1.25 (95% CI 1.18–1.32) and 1.14 (95% CI 1.06–1.22), respectively [20]. Numerous other studies also identify MetS as increasing the risk of CRC [21, 22].

Although the mechanism of MetS in the development of CRC is not clear, it is thought to be related to hyperinsulinism and insulin resistance [23, 24], which increases insulin-like growth factor-1 levels. In addition, adipocyte-secreted hormones such as adiponectin, leptin, and resistin [25]; the greater proportion of Firmicutes and lower proportion of Bacteroidetes within the large intestine [26, 27]; and a high-fat, low-fiber diet [28] are all related to the occurrence of CRC. Since it is important to understand the pathological mechanism of CRC related to MetS [29], additional studies need to examine how to prevent CRC as MetS progresses [30].

In this study, pre-MetS group had a 25% higher risk of CRC and MetS group had a 54% higher risk of CRC than Normal group in men based on the full adjustment model, and pre-MetS group had a 9% higher risk of CRC and MetS group had a 39% higher risk of CRC than Normal group in women. The risk of Mets on incidence of CRC was slightly higher in men than in women, and all were significant.

However, some previous studies showed different risk ratios by gender [10, 12, 31, 32]. The reason for this is difference in the number of study subjects, study design, and fundamental biological differences between males and females. Although this study showed the positive association between MetS and the incidence of CRC, further investigation is needed as to the question of how men and women may be affected by metabolic abnormalities in terms of CRC risk [13].

Lifestyle has been known to be a major factor associated with CRC, and it is known that the risk of cancer gradually decreases if healthy lifestyles are practiced in stages [33]. This study found that drinking alcohol and smoking were related to the risk of CRC. Other studies also shown that smoking [34] and drinking alcohol have carcinogenic effects and promote cancer, especially in the case of drinking alcohol, moderate amounts of drinking alcohol can lead to an increase in CRC [35]. Education programs for lifestyle improvements should be conducted to reduce the incidence of colorectal cancer [36]. It has been suggested as an effective way to raise awareness, knowledge, and screening rate for colorectal cancer screening [37, 38].

There are some studies [39–41] that consider height in the analysis as risk factors for colorectal cancer, and the results of the analysis are also reported to be significant. Based on these similar previous studies, this study considered the relationship between height and colorectal cancer but found no significant correlation. There was also no significant association between weight and colorectal cancer.

Previous studies of Asian races have limits, such as a lack of representativeness or only a few variables were examined. Our study identified MetS as a risk factor for CRC after adjusting for various variables and determined the magnitude of the risk using national health checkup and insurance claims data in Korea. Because there are ethnic differences in the relationship between MetS and cancer [13, 25, 42], the presentation of risk ratios for Asians using large data and long follow-up period sets is a significant research achievement. Study on the relationship between metabolic and disease occurrence for Asian races [43] should be conducted and other approaches to mitigate health risks need to be reviewed [44]. In addition, this study has some newly informative knowledge as follows. First, this study is meaningful in that it attempts to represent the entire Korean population using the National Health Insurance Corporation's claim data. Second, this study is the most recent long-term data taken from the initial establishment of the data source to the latest data. Third, unlike other studies, this study considered the incubation period sufficiently to closely observe the association of colorectal cancer risk due to metabolic syndrome. The incubation period was set to 3 years, and the analysis was performed except for patients with colorectal cancer who developed within 3 years of the observation. Fourth, this study was able to understand the risk of colorectal cancer in more detail by classifying subjects according to the number of factors of metabolic syndrome rather than whether they were metabolic syndrome.

This study has some limitations. First, we did not consider changes in the risk of the number of metabolic components on CRC in men and women in the study population after it was diagnosed, since we used only the health check-up data for 2009. This should be examined in a future study. Second, we did not adjust for some variables, such as the consumption of meat, which is a known risk factor for CRC, because we used secondary data. Lastly, health screening programs are being provided to all Koreans regardless of income, and the participation rate is also increasing from 72.9% in 2012 to 78.5% in 2017 [17]. However, it is mandatory for industrial workers to be screened, and the rate of participation of industrial workers is relatively higher than that of self-employees [45]. Therefore, this study cannot exclude the possibility of health workers effect.

## Conclusions

This study investigated the relationship between MetS and CRC using national health check-up and insurance claims data for Korea. It showed that MetS was a risk factor for the occurrence of CRC.

From a clinical and public health perspective, Mets has emerged as an important disease that requires early management and more thorough management and prevention of Mets are needed to prevent CRC based on the results of this study with long-term follow-up and large-scale of Asian subjects.

## Data Availability

The datasets used in this study are not publicly available due to the limitation of access to the raw data of National Health Insurance Service in Korea. If you need to discuss the dataset, you can e-mail the corresponding author.

## Abbreviations

CIR: Cumulative incidence rates
CRC: Colorectal cancer
ID: Incidence density
MetS: Metabolic syndrome
NCEP-III: National Cholesterol Education Program Adult Treatment Panel III
NHIS: National Health Insurance Service
